# Interleukin 5 Receptor Subunit Alpha Expression as a Potential Biomarker in Patients with Nasal Polyposis

**DOI:** 10.3390/biomedicines11071966

**Published:** 2023-07-12

**Authors:** David Hansoe Heredero-Jung, Sandra Elena-Pérez, Asunción García-Sánchez, Miguel Estravís, María Isidoro-García, Catalina Sanz, Ignacio Dávila

**Affiliations:** 1 Department of Clinical Biochemistry, University Hospital of Salamanca, 37007 Salamanca, Spain; dhheredero.ibsal@saludcastillayleon.es (D.H.H.-J.);; 2 Allergic Disease Research Group IIMD-01, Institute for Biomedical Research of Salamanca (IBSAL), 37007 Salamanca, Spainidg@usal.es (I.D.); 3 Department of Biomedical Sciences and Diagnostics, University of Salamanca, 37007 Salamanca, Spain; 4 Results-Oriented Cooperative Research Networks in Health (RICORS), Carlos III Health Institute, 28029 Madrid, Spain; 5 Department of Medicine, University of Salamanca, 37007 Salamanca, Spain; 6 Department of Microbiology and Genetics, University of Salamanca, 37007 Salamanca, Spain; 7 Department of Allergy, University Hospital of Salamanca, 37007 Salamanca, Spain

**Keywords:** chronic rhinosinusitis, nasal polyposis, CRSwNP, interleukin-5, *IL5RA*, biomarker, gene expression, precision medicine, pharmacogenetics, benralizumab

## Abstract

Chronic Rhinosinusitis with Nasal Polyposis (CRSwNP) affects the quality of life of patients suffering from it. The search for a suitable biomarker has been conducted over the last decades. Interleukin 5 receptor subunit alpha (IL-5Rα) involves the activation, maintenance, and survival of eosinophils, which are highly tied to chronic inflammatory processes of the airways, like asthma or CRSwNP. In this study, we evaluate the utility of *IL5RA* as a genetic biomarker in CRSwNP. *IL5RA* mRNA expression level was analyzed in different groups of patients by performing qPCR assays. A significant increase in *IL5RA* expression was observed in CRSwNP patients, especially those with asthma and atopy. We found differences in expression levels when comparing groups with or without polyposis or asthma, as well as some atypical cases related to eosinophil levels. That opens a path to future studies to further characterize groups of patients with common features in the context of pharmacogenetics and in an era towards developing a more precise personalized treatment with IL-5Rα as a therapeutic target for CRSwNP.

## 1. Introduction

Chronic rhinosinusitis (CRS) is an inflammatory process affecting the paranasal sinuses and the mucosa of the nasal passages, which lasts for 12 weeks or more. Nasal polyposis (NP) is a chronic inflammatory disease encompassing chronic rhinosinusitis. Quality of life (QoL) can be significantly affected in patients with nasal polyposis. Studies by Radenne et al. [1] and Alobid et al. [2], using the Short Form 36 (SF-36), found differences between healthy subjects and NP patients in all items, except for physical function in the case of Alobid et al. In addition, NP patients had greater impairment in the mental sphere than the physical sphere, probably due to the burden that the pathology would cause.

Most CRSwNP patients have a T2 inflammatory disease, with increased tissue and peripheral blood eosinophils. Interleukin (IL)-5 is essential for eosinophil development, differentiation, maturation, and migration. IL-5 activates different target cells, including eosinophils or basophils. It is produced by T lymphocytes (mainly Th2), innate lymphoid cells (ILC2), and mast cells and has effects on proliferation and differentiation through its receptor (IL-5R). The receptor is a heterodimer composed of a ligand-specific α-subunit (IL-5Rα) and the β-subunit, common to other cytokine receptors.

IL-5Rα signal transmission pathways include JAK/STAT, Btk, and Ra without Raf-ERK and involve the maintenance of survival and functionality of B lymphocyte, eosinophil, and basophil populations [3].

The *IL5RA* gene encodes the alpha subunit of the IL-5 receptor. It is located on the short arm of chromosome 3 (3p26.2). A major transcript of this gene, encoding the soluble form of the receptor, was identified from the study in eosinophilic sublines of human eosinophilic promyelocytes and myelocytes cultured from chorionic blood. A second receptor component displays similarities to the β-chain of the high-affinity granulocyte-macrophage colony-stimulating factor receptor (GM-CSFR, *CSF2RB* gene) [4]. *IL5RA* gene expression is regulated by a complex of transcription factors, including E12, E47, Spl1, c/EPBβ, and Oct2.

The membrane isoforms of the IL-5R α-subunit arise from alternative splicing processes, whereas the soluble forms result from a normal splicing process or lack thereof [4].

Recently, new biological drugs have been developed using IL-5 and IL-5Rα as therapeutic targets. Mepolizumab and reslizumab are two examples of humanized monoclonal antibodies against IL-5. Both are approved by the European Medicines Agency (EMA) and the US Food and Drug Administration (FDA) for treating severe eosinophilic asthma [5].

Benralizumab is a humanized monoclonal antibody that binds to the α-chain of IL-5R through its Fab domain, competing against IL-5 binding to its receptor. Through its afucosylated Fc domain, benralizumab binds to the Fc region of the RIIIa receptor of NK cells, macrophages, and neutrophils. Thus, antibody-dependent cellular cytotoxicity targeting eosinophils is induced. Globally, there is an almost complete depletion of eosinophils, reaching medians of 0 eosinophils/μL in peripheral blood in clinical trials [6].

Benralizumab significantly decreased the expression of genes related to eosinophilic inflammatory responses, such as *SIGLEC8*, *ALOX15*, *PRSS33*, *CCL23*, and *IL5RA*. On the other hand, those patients who show a predominant expression of neutrophils-related genes and higher neutrophil counts in peripheral blood have a significantly lower response to benralizumab [7], so basal levels of eosinophils may play a role and must be considered.

The association of T2 asthma with nasal polyposis has been considered a factor predicting response to anti-IL-5 antibodies in asthma [8]. Early studies with anti-IL-5 antibodies such as mepolizumab or reslizumab in patients with CRSwNP reduced polyp size and the need for revision surgery [9]. Studies focusing mainly on CRSwNP and anti-IL-5R therapy were uncommon until recent years. In a trial published in February 2021, Jody et al. suggested a possible positive effect on treating CRSwNP with benralizumab [10]. Efficacy was demonstrated in terms of reduction of polyp size, sinus occupancy, and improvement of symptomatology and olfaction in most patients. This study was, however, limited by its sample size but revealed to some extent, a positive trend of benralizumab effects in CRSwNP. Another study conducted in Osaka [11] reported significant improvements in asthma QoL questionnaires (AQLQ, ACQ5) and respiratory function tests in asthmatic patients with CRSwNP versus patients with asthma without polyposis.

Benralizumab is currently approved for the treatment of asthma [12]. The phase III results for the efficacy of benralizumab in CRSwNP (OSTRO) reported a reduction in nasal blockage and nasal polyp scores [13]. There are other biological treatments currently used or under evaluation for CRSwNP. Omalizumab, mepolizumab, and dupilumab were approved for CRSwNP in the US and Europe. Dupilumab was approved for the treatment of CRSwNP [14], being the first biological therapy approved for treating adults with inadequate control of CRSwNP in both the European Union and the United States. Omalizumab has demonstrated safety and efficacy in the treatment of CRSwNP after 52 weeks of treatment [15], improving quality of life (QoL) scores, suggesting an even greater benefit as treatment is prolonged (52 weeks vs. 24) and a reduced need for nasosinusal surgery in the future. Recent results from the SYNAPSE trial with mepolizumab [16] have also been encouraging for the quality of life of patients with CRSwNP, compared to placebo, with higher effectiveness in patients with associated asthma or N-ERD, but with similar behavior in those individuals without comorbidities. Fevipiprant has recently failed the established objectives, but it may remain appealing in the study of well-characterized groups of patients [17].

As mentioned, CRSwNP has been considered an indicator to predict the response to anti-IL-5 antibodies in T2 asthma. IL-5 antagonist therapy is helpful in patients with CRSwNP. Therefore, the main objective of this study is to determine the levels of *IL5RA* gene expression in peripheral blood in a population of patients with nasal polyposis versus a control group and to analyze the differences in expression among different clinical phenotypes, and the possibility of considering *IL5RA* a suitable biomarker for nasal polyposis, with regards to a more precise approach to the treatment of CRSwNP.

## 2. Materials and Methods

Peripheral blood samples were obtained from 256 individuals from the Allergy Department of the University Hospital of Salamanca. All patients signed an informed consent form. The legal regulations for clinical trials in Spain and the authorization of the Committee on Drug Research Ethics (CEIm, PI 2020-02-433) were obtained.

Controls were selected according to the following inclusion criteria: (i) absence of symptoms and personal history of allergy; (ii) negative skin tests against a battery of aeroallergens common in our environment (mites, epithelia, fungi, pollens); (iii) absence of symptoms and personal history of asthma; (iv) absence of symptoms and personal history of other respiratory pathologies; (v) absence of a family history of atopy, asthma or allergic rhinitis; and (vi) age over 16 years old. The experimental group included patients presenting allergic respiratory symptomatology, diagnosed with chronic rhinosinusitis with nasal polyposis, whether accompanied by asthma or not, and those diagnosed with positive and negative skin tests (allergic and nonallergic asthma).

### 2.1. Study Variables

Variables were collected in a structured questionnaire at the consultation, and analytical and clinical data were obtained from the laboratory informatics system (LIS) and patients’ medical records. Variables included in the questionnaire comprised the following: patient demographics; positivity or not of skin prick tests; diagnosis of asthma rhinitis, and polyposis; age of onset of symptoms; presence or not of hypersensitivity to NSAIDs; as well as the existence of a family history, immunotherapy, or corticosteroid treatment.

The diagnostic criterion for atopy was positivity to at least one allergen of a battery of skin prick tests against mites, peptides, fungi, and pollens. Skin tests were performed according to the recommendations for allergen standardization and skin testing issued by the European Academy of Allergy and Clinical Immunology (EAACI). The aeroallergen battery (ALK-Abelló, Madrid; Bial-Aristegui, Bilbao; CBF-Leti, Barcelona) was adapted to local exposure. Patients with positive skin prick tests were considered atopic. CRSwNP was diagnosed according to EPOS 2020 criteria [18], and asthma according to GEMA guidelines [19] and subsequent guidelines. Allergic asthma (AA) was considered in patients with positive skin tests and symptoms compatible with exposure to the sensitizing allergen, and nonallergic asthma (NAA) was asthma with negative skin prick tests. Phenotype classification of asthma according to the presence of eosinophilia, FeNO, and atopy was also considered (Spanish Guide for the Management of Asthma—GEMA 5.1 [19]), being asthma type 2 (T2) characterized by total peripheral blood eosinophil level ≥ 150/µL or FeNO ≥ 25 ppm, or presence of allergy. In contrast, non-type 2 (non-T2) asthma should have a peripheral blood total eosinophil level < 150/µL and FeNO < 25 ppm and absence of allergy. FeNO was determined using a portable device (Niox Vero, Circassia, Oxford, UK). Rhinitis was diagnosed based on ARIA (Allergic Rhinitis and its Impact on Asthma) 2019 guidelines [20].

Total IgE levels were determined by enzyme immunoassay in an ImmunoCAP 250 (Thermo Fischer Scientific, Waltham, MA, USA) using the ImmunoCAP Total IgE kit, and blood cells count was determined by flow cytometry on a Sysmex XN-1000 system (Roche Diagnostics, Rotkreuz, Switzerland).

### 2.2. qPCR Expression Assays

The RiboPure™-Blood RNA Purification kit (Ambion, Thermo Fisher Scientific, Waltham, MA, USA) was used for mRNA extraction and purification. Samples were subjected to an additional purification process with DNase I. RNA later solution was used to store samples before extraction. Both reagents were included in the kit.

qPCR was performed on a LightCycler^®^ 480 (Roche Diagnostics, Switzerland). The mRNA expression of IL5RA was determined using the LightCycler^®^ 480 SYBR Green I Master (Roche Diagnostics, Switzerland). GAPDH and TBP were used as reference genes. Identifying optimal regions in the gene for primer design was performed using Primer3web and Beacon Designer web tools from Premier Biosoft. Table 1 lists the primers used in the study. Primers were designed to amplify a coding DNA region (cDNA) for each gene, thus avoiding the amplification of genomic DNA, so some of the oligonucleotides are designed at the exon–exon junction, as is the case of the reverse primer of IL5RA. The designed primers were ordered and manufactured by Integrated DNA Technologies, IDT (Coralville, IA, USA). The oligonucleotides for the reference genes, GAPDH and TBP, are commercial designs (Human Reference Gene Panel, Roche Diagnostics, Switzerland). The efficiency of the primers was studied with serial dilutions amplifications, and all of them ranged from 90 to 110% (Efficiency = (10^−1/slope^ − 1) × 100).

The calibrator used was obtained from commercial total RNA from peripheral blood lymphocytes of 426 individuals of Asian ethnicity (men and women aged 18–54 years) (Human Blood, Peripheral Leukocytes Total RNA, Takara, Kyoto, Japan). Total RNA from the samples and calibrator was retrotranscribed to cDNA using the SuperScript^®^ III First-Strand Synthesis System kit (Thermo Fisher Scientific, Waltham, MA) using random hexamers. The qPCR mix for each well contained 15 µL (7.5 µL of SYBR Green Master Mix, 1.3 µL of 10 µM primer mix, 1.2 µL of distilled water, and 5 µL of sample solutions with 20 ng of cDNA). Each reaction was performed in triplicate, and non-template controls (distilled water) were included in each assay. The conditions of the qPCR were as follows: (i) 1 pre-incubation cycle of Taq Polymerase activation at 95 °C for 5 min 4.4 °C/s ramp; (ii) 45 amplification cycles, each consisting of 10 s at 95 °C (denaturation, 4.4 °C/s ramp), 10 s at 60 °C (annealing, 2.2 °C/s ramp) and 10 s at 72 °C (polymerization, 4.4 °C/s ramp); (iii) 1 cycle for the melting curve, 5 s at 95 °C (4.4 °C/s ramp), 1 min at 65 °C (2.2 °C/s ramp) and target temperature of 97 °C; and the cooling cycle to 40 °C in 30 s (2.2 °C/s ramp). The individual melting curves were analyzed, expression levels of IL5RA were normalized against the reference genes, and expression calculation was performed using the comparative method of ΔΔCt [21]. All qPCR expression calculation methods followed the recommendations of the MIQE guidelines (Guide to Performing Relative Quantification of Gene Expression Using Real-Time Quantitative PCR [22]).

### 2.3. Statistical Analyses

The results were analyzed using Excel spreadsheets (Office 365 v.2306; Microsoft, Redmond, Washington, DC, USA) and SPSS Statistics v.15 and 17 (IBM, Endicott, New York, NY, USA), GraphPad Prism 6 (GraphPad, San Diego, CA, USA) and R v4.2.1 (R Foundation for Statistical Computing, Vienna, Austria). Packages used included ggplot2, tidyverse, fastDummies, heatmaply, pROC, dplyr, and skimr [23,24,25,26,27,28,29]. Kolmogorov–Smirnov Z was used to analyze the normality of the distribution; Levene’s test for the study of homogeneity of variances; ANOVA and Kruskall-Wallis were used for the comparison of means for normal and non-normal distributions, respectively; and Pearson correlation was used to measure the degree of linear dependence between 2 continuous quantitative variables and to generate the correlation plots.

## 3. Results

### 3.1. Demographic Data

The study sample comprised 256 individuals; 102 (39.85%) belonged to the control group and 154 (60.15%) to the experimental group. Demographic characteristics are shown in Table 2.

All patient groups had significantly higher eosinophil levels than controls (*p* < 0.001). The difference between CRSwNP with asthma and CRSwNP without asthma and between CRSwNP with AA and CRSwNP with NAA were non-significant. The NAA without NP group showed the lowest levels of eosinophils besides controls. IgE levels were also significantly higher in the patients’ groups than in the controls (*p* < 0.001). The highest mean concentration was that of the CRSwNP with the AA group (609.35 ± 924.31).

### 3.2. IL5RA Expression Analysis

**Figure 1:**
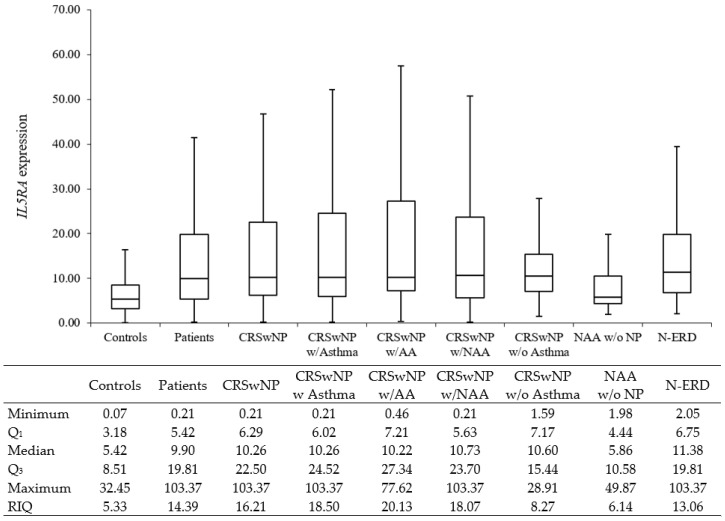
Boxplots of *IL5RA* expression in peripheral blood vs. *GAPDH*. CRSwNP: chronic rhinosinusitis with nasal polyposis. AA: allergic asthma. NAA: nonallergic asthma. N-ERD: NSAIDs Exacerbated Respiratory Disease. w/: with. w/o: without.

Table 3 shows the differences in the relative expression of *IL5RA* amongst all groups. Compared to controls, almost every group of patients showed a statistically significant increase in *IL5RA* expression levels (*p* < 0.001). The only group that did not reach significance was NAA without NP. Similar relative *IL5RA* expression was observed with *GAPDH* and *TBP* as reference genes. Therefore, only relative *IL5RA* expression values against *GAPDH* will be considered from now on due to its wide use in the scientific literature and similar papers. The median *IL5RA* expression of the overall patient sample was 9.9 ± 14.4 (median ± RIQ), and the median of the control group was 5.42 ± 5.33. Those groups with higher median expression levels were CRSwNP without atopy (11.4 ± 17.5) and N-ERD (11.4 ± 13.1). Boxplots from Figure 1 show a clear difference in expression levels between groups of patients and controls.

Statistically significant differences were found between *IL5RA* expression levels between CRSwNP (n = 121) and controls (*p* < 0.001), with medians of 10.3 ± 16.2 and 5.42 ± 5.33, respectively. A significant increase in *IL5RA* expression levels was also observed in the group of CRSwNP with atopy (n = 49) when compared with controls (*p* < 0.001), with a median expression of 10.1 ± 11.3 in atopic patients. The non-atopic patients with CRSwNP also presented a statistically significant increase in expression levels (11.4 ± 17.5, *p* < 0.001).

Considering asthma, CRSwNP patients, with or without concomitant asthma, showed higher *IL5RA* expression than controls (10.3 ± 18.5 and 10.6 ± 8.26, respectively, *p* < 0.001), as well as both CRSwNP with AA and CRSwNP with NAA groups (10.2 ± 20.1 and 10.7 ± 18.1, respectively, *p* < 0.001). However, in the subgroup of patients with NAA, which had no nasal polyposis (NAA without NP), the elevation of expression levels did not reach significance (*p* = 0.088), with a median of 5.86 ± 6.14.

Interestingly, there were no significant differences between CRSwNP with asthma and CRSwNP without Asthma (*p* = 0.329) and between CRSwNP with AA and CRSwNP with NAA (*p* = 0.411). As for the difference when comparing the CRSwNP with NAA expression levels and those from the NAA without NP group did not reach significance, but a low *p*-value was obtained (*p* = 0.081). The main difference between these two groups roots in the presence or absence of NP.

### 3.3. Correlation between IL5RA Expression, Eosinophil Count, and Total IgE Levels

There was a significant positive correlation (*p* < 0.001) between *IL5RA* expression levels and peripheral blood eosinophil count, as well as with IgE levels in peripheral blood (*p* < 0.001). Table 4 shows the correlation between IgE and eosinophils (Pearson, r) by subgroups. Table 5 shows the correlation against different cell lines’ counts, with a significant positive correlation with eosinophils, basophils, and the sum of both.

**Figure 2:**
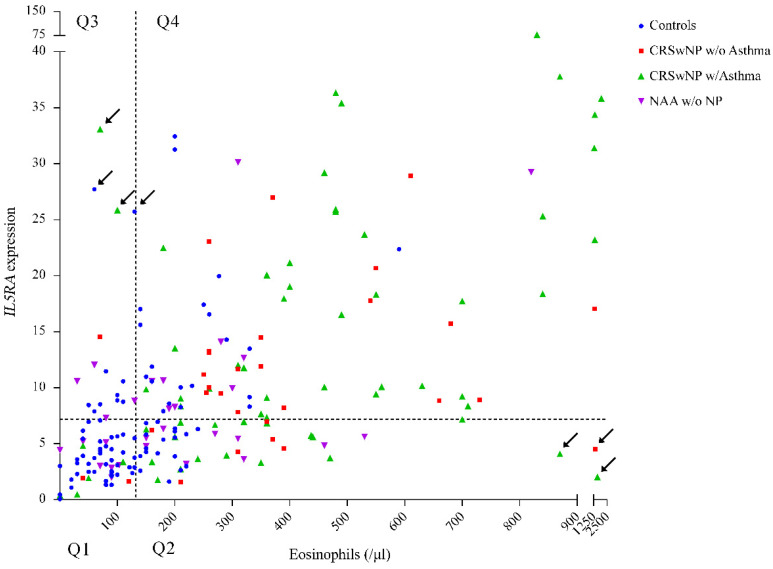
Scatter plot of *IL5RA* expression and peripheral blood eosinophil count (EOS) (cells/µL). Calculations were performed using the comparative ΔΔC method. All procedures followed the recommendations of MIQE guidelines. Q1: quadrant 1 (EOS ≤ 132.43; *IL5RA* ≤ 7.2); Q2: quadrant 2 (EOS > 132.43; *IL5RA* ≤ 7.2); Q3: quadrant 3 (EOS ≤ 132.43 *IL5RA* > 7.2); Q4: quadrant 4 (EOS > 132.43; *IL5RA* > 7.2). The cutoff points correspond to the mean values in controls for both variables. CRSwNP: chronic rhinosinusitis with nasal polyposis; A: asthma; NAA: nonallergic asthma. Arrows point to outliers.

The following scatter plot (Figure 2) shows the distribution of *IL5RA* expression levels against eosinophil count in peripheral blood. It is interesting to point out some outliers in the plot (marked with an arrow), corresponding to patients with low eosinophil levels but with high *IL5RA* expression (and vice versa).

**Figure 3:**
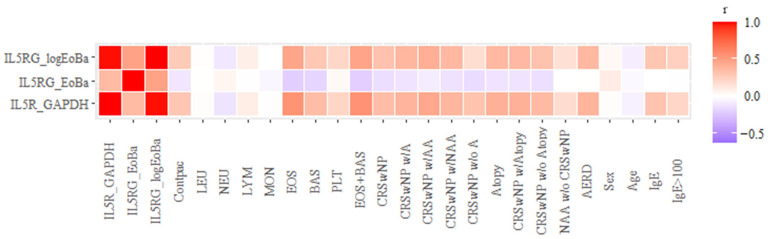
Colorimetric correlation matrix. A negative correlation is shown in blue, and a positive correlation is in red. The intensity of the color corresponds to the degree of correlation (correlation coefficients (Pearson) closer to −1 and 1). White color denotes correlation coefficients close to 0. *IL5R_GAPDH*: expression or *IL5RA* using *GAPDH* as the reference gene. Contpac: control = 0, patient = 1. LEU: peripheral blood leukocyte concentration. NEU: neutrophils. LYM: lymphocytes. MON: monocytes. EOS: eosinophils. BAS: basophils. PLT: platelets. IgE: IgE concentration in kU/L. IgE > 100: IgE concentration greater than 100 kIU/L.

Figure 3 shows a correlation matrix with all the study variables. Qualitative variables were expressed in binary terms as dummy variables (0 = absence of the condition; 1 = presence of the condition). Variables were added from the normalization of *IL5RA* expressions against *GAPDH*, with the sum of eosinophil plus basophil concentrations and the logarithms of these sums. The aim was to minimize the influence of eosinophil and basophil concentrations in peripheral blood (cells where the *IL5RA* gene is expressed to a greater extent) when studying the increased expression of this gene among the different groups of patients. The correlation of normalized expression with the logarithm of the concentration of eosinophils plus basophils is maintained with respect to unnormalized gene expression, as can be seen by the color coding of the matrix. However, it is lost in some clinical groups when dividing the expression by the raw concentration.

## 4. Discussion

Biomarkers most frequently employed in the diagnosis of CRS can be obtained from various biological fluids and secretions, with peripheral blood being the most accessible and requiring the least time and expertise. However, peripheral blood does not always reflect the local inflammation and nasal microenvironment. Nasal secretions obtained by lavage also have diagnostic utility, although they may not always correspond to the local inflammation and microenvironment and have reproducibility issues because of the lack of homogeneity when making dilution calculations.

The very ease of obtaining the sample ensures, to a certain extent, a greater reproducibility of the study, giving consistency and robustness to the technique. Peripheral blood meets this accessibility criterion, which is one reason the present study has been conducted to discern the differences in *IL5RA* expression in CRSwNP patients. Many CRS biomarkers arise from proof-of-concept studies of other related disease markers, such as asthma, atopic dermatitis, or environmental allergies. Such is the case of *IL5RA.*

As for *IL5RA* expression for CRSwNP, the ease and accessibility to obtain the sample from peripheral blood, a minimally invasive method for the patient, outperforms more complex specimens, such as induced sputum in the case of asthma or biopsy in the case of CRSwNP. Although it is not as simple and automated as determining eosinophils in peripheral blood, RNA expression techniques have been greatly simplified [30]. This biomarker is suited to reflect the etiopathogenic mechanisms of CRSwNP. In this regard, nasal polyposis is associated with tissue and peripheral blood eosinophilia.

As expression assays were performed in triplicate, the mean value and deviation were considered (along with intra-assay variability acceptance criteria), minimizing the effect of random error. Furthermore, the RNA later solution used to store the samples is intended to ensure the stability of RNA in whole blood. It allows samples to be stored for up to 3 days at room temperature or long-term at −20 °C. It maintains the gene expression profile of the cells so that the RNA obtained at the end of the extraction is comparable to that obtained if the samples were processed directly without storage. In addition, the reagent significantly reduces genomic contamination of the samples.

In general terms, median levels of *IL5RA* expression in patients were almost twice as high as those in controls, being more pronounced in patients with CRSwNP only, with a peak in those with allergic asthma associated (almost threefold). This data is relevant since it reflects its capacity to distinguish between patients and controls. In addition, differences in expression levels should be analyzed to evaluate the performance of the biomarker to distinguish different clinical situations, such as asthma, respiratory allergy, or N-ERD. In this regard, we observed the highest expression levels in those patients combining CRSwNP, asthma, and atopy. However, no statistically significant differences were found between the main groups. It should be considered, nevertheless, that the comparison between patients with nonallergic asthma without nasal polyposis and controls showed quite close medians and the difference was not significant. This behavior could help distinguish between this type of asthma (probably non-T2) and typical T2 asthma. However, the small size of this subgroup should be considered.

Due to the non-normal distribution of the variables, it was necessary to perform the logarithmic transformation of the concentration of eosinophils plus basophils for correlation studies. The correlation is maintained with respect to that of the unnormalized expression. This data further supports the predicted activity of *IL5RA*, as the influence of the concentrations of these cell populations is minimized.

In the literature, N-ERD patients have already shown a relationship between the development of nasal polyps and *IL5RA* levels [31]. In our study, the difference in expression levels is maintained significantly, despite the small sample size.

There was a correlation between eosinophil levels and *IL5RA* expression in peripheral blood. However, this correlation, although significant, was weak. As we can see, some patients may show high expression levels but a not-so-high eosinophil count. Flow cytometry studies, marking IL-5Rα [31], could prove this scenario and give the gene expression an independent role from other biomarkers in CRSwNP, such as eosinophils. Thus, when comparing *IL5RA* expression with eosinophil count, we can observe some atypical cases with high gene expression despite not having eosinophilia. Remarkably, there are also patients with high levels of eosinophils and low expression of *IL5RA.* Those two populations of patients suggest that the correlation between eosinophils and *IL5RA* expression levels is not linear, and it raises the possibility that, at least in some patients, the discordance could help select treatments. Whether patients with low eosinophil levels and high *IL5RA* expression levels would benefit from treatment with benralizumab or, the other way around, whether patients with high eosinophil count and low expression would respond to this makes for an exciting topic to study in more depth.

Also notable is the lack of correlation between *IL5RA* expression and eosinophils in the CRSwNP group without asthma when the differences in expression between this group and controls were significant. In contrast, the opposite occurs in the NAA without NP group: the correlation between *IL5RA* and eosinophils was significant, whereas the difference in expression versus controls was not. That would also support the independence between both biomarkers, at least in some populations. The gene expression and the basophil count were also significantly correlated, as would be expected from a physiological point of view.

No statistically significant differences were found between expression levels in CRSwNP patients with asthma and those without asthma, but it was between both groups and controls, suggesting that the weight of the difference in expression in patients with NP may not be due to the presence of asthma. No significant differences were also found between expression levels in CRSwNP with AA and CRSwNP with NAA, indicating that allergy does not seem to act as a differential factor in *IL5RA* expression. The difference in expression levels observed between patients with nonallergic asthma-associated NP (CRSwNP with NAA) and those with nonallergic asthma without associated nasal polyposis (NAA without NP) is worth mentioning. This would reinforce that NP could be responsible for the increased expression of the *IL5RA* gene. Indeed, a *p*-value close to significance was obtained. This datum points to a greater weight of NP in the difference in expression compared to atopy in asthma.

The time of asthma onset, although not taken into account in our study, would be interesting to consider for evaluation of the response to treatment with biologics, as described in the literature [32]

Although this article has focused on the study of peripheral blood expression of IL5RA, in future studies, it would be interesting to analyze in parallel the expression of the membrane-anchored expression (TM-IL-5Rα) isoform in peripheral blood and in nasal tissue from control subjects and nasal polyp (NP) patients, since different expression levels have been previously described for both isoforms [33].

IL-5 is an essential cytokine in eosinophil production, maturation, migration, and activation [34]. A monoclonal treatment specifically targeting IL-5Rα, benralizumab, has already been developed, so determining *IL5RA* expression may help evaluate the response. Whether or not the determination of the expression of *IL5RA* would help predict which patients would respond better to benralizumab makes for an exciting field to explore, and it has already been reviewed in a recent study from our research group, which seems to support this idea in so far as *IL5RA* expression relates, to some extent, to the response to benralizumab and the improvement of FEV_1_ and the asthma control test scores (ACT) [34]. Nakajima et al. [7] identified a group of super-responders that showed a significant reduction of *IL5RA* post-treatment (among other genes associated with eosinophilic inflammation).

Treatment with benralizumab seemed to have a future projection in CRSwNP, as showed some data from patients with asthma and CRSwNP [35,36,37]. A Phase II study conducted in Japan [38] showed a reduction in Nasal Polyp Scores after treatment with benralizumab, especially in patients with high starting levels of peripheral blood eosinophils, although benralizumab did not meet the primary efficacy point. Also, in the OSTRO pivotal trial of benralizumab in patients with CRSwNP, the biological decreased Nasal Polyp Score by 0.570 (*p* < 0.001) and Nasal Blockage Score by 0.270 (*p* < 0.005) but not statistically significant between-group differences were observed at the time of first nasal polyp surgery or Sinonasal Outcome Test-22 (SNOT-22). The authors stated, “CRSwNP is a heterogeneous disease that includes subsets of patients for whom eosinophils may be a more significant contributor to disease pathogenesis as well as patients for whom other inflammatory mediators or alternative pathways are driving the formation and persistence of nasal polyps” [13]. It is possible that determining *IL5RA* expression levels could help distinguish these subsets of patients.

## 5. Conclusions

The present results support the role of *IL5RA* as a biomarker in CRSwNP. As *IL5RA* levels in peripheral blood do not always correlate with peripheral blood eosinophil levels, its determination could provide further information to that provided by eosinophils. Of course, as this is a first approach, further studies are needed to identify those possible groups of patients who would be more susceptible to developing nasal polyposis and its progression, predicting drug response or surgical treatment needs. The customization of clinical management and the individualization of each specific case will allow us to obtain the optimal treatment for each patient in a new pharmacogenetic era based on precision medicine.

## Figures and Tables

**Table 1:** Primer sequences used in the qPCR assays for *IL5RA*, *GAPDH*, and *TBP* (GRCh38/hg38).

Gene	Sense	Sequence
*IL5RA*	Forward	5′-TGAAAGAGTGAAGAACCGCC-3′
	Reverse	5′-CCTGGCCTGAGAAATGCG-3′
*GAPDH*	Forward	5′-CTCTGCTCCTCCTGTTCGAC-3′
	Reverse	5′-ACGACCAAATCCGTTGACTC-3′
*TBP*	Forward	5′-GAACATCATGGATCAGAACAACA-3′
	Reverse	5′-ATAGGGATTCCGGGAGTCAT-3′

**Table 2:** Demographic characteristics and IgE and peripheral blood eosinophil levels of the study sample.

	n	Sex ^†^	Age ^1^	IgE ^1^	Eosinophils ^1^
Controls	102	0.67	56.97 ± 17.43	68.41 ± 113.96	132.43 ± 90.4
Patients	154	0.47	54.25 ± 16.13	289.71 ± 556.85	444.23 ± 366.45
CRSwNP	121	0.40	53.86 ± 16.16	329.71 ± 602.5	510.02 ± 382.9
CRSwNP with Atopy	49	0.31	46.6 ± 15.26	526.72 ± 836.79	524.81 ± 307.57
CRSwNP without Atopy	72	0.46	58.69 ± 14.98	191.2 ± 293.34	499.76 ± 429.61
CRSwNP with Asthma	87	0.45	54.17 ± 15.61	400.05 ± 684.57	547.69 ± 403.02
CRSwNP with AA	38	0.37	46.57 ± 15.53	609.35 ± 924.31	561.68 ± 323.65
CRSwNP with NAA	49	0.51	59.92 ± 13.13	233.56 ± 332.45	536.1 ± 426.36
CRSwNP without Asthma	34	0.27	53.06 ± 17.7	144.5 ± 206.1	415.83 ± 313.77
NAA without NP	33	0.73	55.7 ± 16.19	149.04 ± 321.92	221.39 ± 171.93
N-ERD	26	0.50	57.31 ± 12.57	304.81 ± 347.67	503.18 ± 386.98

^1^ Results expressed as mean ± SD. n, number of cases. ^†^ Female sex (percentage). IgE (kU/L). Eosinophils (concentration expressed in cells/µL). CRSwNP: chronic rhinosinusitis with nasal polyposis. AA: allergic asthma. NAA: nonallergic asthma. N-ERD: NSAIDs Exacerbated Respiratory Disease.

**Table 3:** Results of contrast studies of *IL5RA* expression. Kruskal–Wallis test (*p*). Calculations were performed using the comparative ΔΔCt method. All procedures followed the recommendations of MIQE guidelines [30]. n = number of cases; IQR = interquartile range. CRSwNP: chronic rhinosinusitis with nasal polyposis. AA: allergic asthma. NAA: nonallergic asthma. N-ERD: NSAIDs Exacerbated Respiratory Disease.

		*IL5R/GAPDH*			*IL5R/TBP*		
	n	Median	IQR	*p*	Median	IQR	*p*
Controls	102	5.42	5.33	*-*	1.68	2.22	*-*
Patients	154	9.9	14.4	<0.001	3.48	4.81	<0.001
CRSwNP	121	10.3	16.2	<0.001	4.12	6.55	<0.001
CRSwNP with Atopy	49	10.1	11.3	<0.001	4.73	5.9	<0.001
CRSwNP without Atopy	72	11.4	17.5	<0.001	3.88	4.97	<0.001
CRSwNP with Asthma	87	10.3	18.5	<0.001	4.12	6.55	<0.001
CRSwNP with AA	38	10.2	20.1	<0.001	4.92	7.49	<0.001
CRSwNP with NAA	49	10.7	18.1	<0.001	3.49	5.59	<0.001
CRSwNP without Asthma	34	10.6	8.26	<0.001	4.15	3.34	<0.001
NAA without NP	33	5.86	6.14	0.088	2.15	2.04	0.36
N-ERD	26	11.4	13.1	<0.001	4.04	5.42	<0.001

**Table 4:** Correlations by groups. *IL5RA* expression levels versus serum IgE levels and peripheral blood eosinophil count. Correlation (Pearson, r). Significant correlations are highlighted in bold. EOS: eosinophil count.

	n	*IL5RA* and IgE	*IL5RA* and EOS
Controls	102	−0.1 (*p* = 0.339)	**0.46 (*p* < 0.001)**
Patients	154	**0.28 (*p* < 0.001)**	**0.49 (*p* < 0.001)**
CRSwNP	121	**0.26 (*p* = 0.007)**	**0.47 (*p* < 0.001)**
CRSwNP with Atopy	49	**0.38 (*p* = 0.011)**	**0.54 (*p* < 0.001)**
CRSwNP without Atopy	72	0.21 (*p* = 0.092)	**0.44 (*p* < 0.001)**
CRSwNP with Asthma	87	**0.27 (*p* = 0.017)**	**0.49 (*p* < 0.001)**
CRSwNP with AA	38	**0.36 (*p* = 0.036)**	**0.52 (*p* = 0.002)**
CRSwNP with NAA	49	0.21 (*p* = 0.182)	**0.48 (*p* = 0.002)**
CRSwNP without Asthma	34	−0.01 (*p* = 0.952)	0.18 (*p* = 0.333)
NAA without NP	33	0.31 (*p* = 0.085)	**0.48 (*p* = 0.007)**
N-ERD	26	**0.67 (*p* < 0.001)**	0.31 (*p* = 0.165)

**Table 5:** Correlations between *IL5RA* expression levels and blood cell count (cells/µL). EOS + BAS: sum of eosinophils and basophils.

		Neutrophils	Lymphocytes	Monocytes	Eosinophils	Basophils	Eos + Bas
*IL5RA* (vs. *GAPDH*)	r	−0.115	0.096	0.008	0.55	0.343	0.557
	*p*	0.082	0.147	0.903	<0.001	<0.001	<0.001

## Data Availability

The data presented in this study are available on request from the corresponding author. The data are not publicly available due to privacy.
